# Dangerous Pathogens as a Potential Problem for Public Health

**DOI:** 10.3390/medicina56110591

**Published:** 2020-11-06

**Authors:** Edyta Janik, Michal Ceremuga, Marcin Niemcewicz, Michal Bijak

**Affiliations:** 1 Biohazard Prevention Centre, Faculty of Biology and Environmental Protection, University of Lodz, Pomorska 141/143, 90-236 Lodz, Poland; edyta.janik@unilodz.eu (E.J.); marcin.niemcewicz@biol.uni.lodz.pl (M.N.); 2 Military Institute of Armament Technology, Prymasa Stefana Wyszyńskiego 7, 05-220 Zielonka, Poland; ceremugam@witu.mil.pl

**Keywords:** public health, pathogens, biothreats

## Abstract

Pathogens are various organisms, such as viruses, bacteria, fungi, and protozoa, which can cause severe illnesses to their hosts. Throughout history, pathogens have accompanied human populations and caused various epidemics. One of the most significant outbreaks was the Black Death, which occurred in the 14th century and caused the death of one-third of Europe’s population. Pathogens have also been studied for their use as biological warfare agents by the former Soviet Union, Japan, and the USA. Among bacteria and viruses, there are high priority agents that have a significant impact on public health. *Bacillus anthracis*, *Francisella tularensis*, *Yersinia pestis*, Variola virus, Filoviruses (Ebola, Marburg), Arenoviruses (Lassa), and influenza viruses are included in this group of agents. Outbreaks and infections caused by them might result in social disruption and panic, which is why special operations are needed for public health preparedness. Antibiotic-resistant bacteria that significantly impede treatment and recovery of patients are also valid threats. Furthermore, recent events related to the massive spread of Severe acute respiratory syndrome coronavirus 2 (SARS-CoV-2) are an example of how virus-induced diseases cannot be ignored. The impact of outbreaks, such as SARS-CoV-2, have had far-reaching consequences beyond public health. The economic losses due to lockdowns are difficult to estimate, but it would take years to restore countries to pre-outbreak status. For countries affected by the 2019 coronavirus disease (COVID-19), their health systems have been overwhelmed, resulting in an increase in the mortality rate caused by diseases or injuries. Furthermore, outbreaks, such as SARS-CoV-2, will induce serious, wide-ranging (and possibly long-lasting) psychological problems among, not only health workers, but ordinary citizens (this is due to isolation, quarantine, etc.). The aim of this paper is to present the most dangerous pathogens, as well as general characterizations, mechanisms of action, and treatments.

## 1. Introduction

Pathogens are defined as organisms that can cause diseases in their hosts. They are taxonomically widely diverse, and include bacteria, viruses, unicellular and multicellular eukaryotes, and are prevalent in the environment [1]. Many pathogens can be transmitted from the environment to the human population, cause outbreaks, and become a threat to public health. Table 1 summarizes the most important and dangerous representatives of pathogens. One example is *Vibrio cholerae*, a natural inhabitant of aquatic ecosystems transmitted by contaminated water or food, causing cholera. *Campylobacter jejuni* is a causative agent of bacterial gastroenteritis, which grows in the mammalian and avian intestinal tracts and is transmitted through contaminated milk, poultry, and water. *Legionella pneumophila* is found in streams, ponds, and plumbing installations. It is the cause of Legionnaires’ disease, and infection occurs by inhalation of aerosols [2]. Soil is also a habitat for bacteria. One of many examples is *Bacillus cereus*, which, besides soil, occurs in the rhizosphere of some plants, and can cause a foodborne disease (emetic and diarrheal illness). Spores of *Bacillus anthracis*, an etiological agent of anthrax, can survive in soil for decades. During World War II, the British government conducted an experiment with the use of *B. anthracis* on Gruinard Island. This operation resulted in soil contamination with anthrax spores for almost 50 years [3,4].

Laboratory studies prove that influenza A viruses (IAVs) may remain contagious in water and sediments for weeks or even months. Several studies have reported the isolation of IAVs from beach sand and surface water, from places of residence of wild birds, which are hosts of IAVs [28]. For centuries, epidemics occurred as a consequence of the action of pathogens and have played a significant role in the advancement of human civilization. The first outbreak of a plague, known as the Plague of Justinian, lasted between 541 and 542 AD. During the epidemic, roughly 100 million Europeans and 40% of habitants of Constantinople died. The second outbreak, the Black Death, occurred in the 14th century and led to the death of 25 million people, which was one-third of the population of Europe. The pneumonic plague epidemic, in the years 1910–1911 in Northern China, is described as the worst epidemic of the 20th century, as it took more than 60,000 lives [29,30]. In the 21st century, plagues are present in Africa, Asia, and the Americas. More than 90% of disease cases appear in Africa, especially in Madagascar, Democratic Republic of Congo, and Uganda [31]. 

Smallpox caused by *Variola virus* is recognized as the most sustained and widespread pandemic in history, with a high mortality rate [32]. Throughout the 18th century, smallpox reduced the human population. Disease occurred in the United States of America, Great Britain, Sweden, and Finland. In the 20th century, smallpox led to a large number of deaths in Europe, North America, Asia, and Africa. Despite a vaccine developed by Edward Jenner at the end of 18th century, smallpox accounted for some 10–15 million cases annually until 1967. In 1980, the disease was considered eradicated after a successful global vaccination program [33,34,35]. Some pathogens are considered as potential candidates for use in armed conflicts as biological warfare agents. The most well-known case of using anthrax as a bioterrorism agent was the mailing of letters containing spores of *Bacillus anthracis*, after a terrorist attack in 2001 [36]. Moreover, during World War I, the German army performed research on cholera and anthrax as bioweapons. The Japanese government in Manchuria, at a place known as Unit 731, conducted military research on many different biological agents. Unit 731 performed tests on prisoners with diseases, such as anthrax, the plague, cholera, and yellow fever [37,38]. During the Cold War, the Soviet Union enlarged its biological weapons program, named “Biopreparat”. Huge amounts of biological agents were produced and stored. The program comprised of research of anthrax, the plague, smallpox, glanders, tularemia, brucellosis, Marburg virus, and Venezuelan equine encephalitis virus. Aspects, such as production, genetic modifications, and weaponization were studied [39]. The Centers for Disease Control and Prevention (CDC) categorized agents (that can be used as biological weapons) into groups. Among the most dangerous pathogens are: *Bacillus anthracis*, *Yersinia pestis*, *Francisella tularensis*, Ebola virus, Marburg virus, Variola major, and Lassa virus [40]. Pathogens undoubtedly constitute a serious threat to human health and life, which is why rapid identification of pathogenic organisms and appropriate treatments are important. Polymerase chain reaction (PCR), flow cytometry, bioluminescent sensors, optical biosensors, enzyme-linked immunosorbent assay (ELISA), or colony counting and culturing are currently used [41]. 

The aim of this paper is to introduce the most dangerous pathogens. General characterization, mechanisms of action, and treatments are presented.

## 2. Bacterial Pathogens with High Impact Potential on Public Health

### 2.1. Bacillus anthracis

*Bacillus anthracis* is a Gram positive, non-motile, facultative anaerobic, spore forming, and rod-shaped bacterium, which belongs to the *Bacillus cereus* sensu lato group. This group is shared by seven species, including *B. cereus*, *B. thuringiensis*, *B. mycoides*, *B. pseudomycoides, B. cytotoxicus, B. toyonensis, and B. weihenstephanensis.* The mentioned species are divergent, phenotypically and pathogenically [42,43]. *Bacillus anthracis* is an etiological agent of anthrax, a zoonotic—currently a rare disease in humans that is associated with potential bioterror use, and considered as a serious biological threat. Annual estimates of cases worldwide range from 20,000 to 100,000, and it is also regarded as a major threat to public health in central Asia, Haiti, Africa, South America, and the Middle East. *B. anthracis* is an obligate pathogen, because bacterial reproductive cycles occur only in a suitable host. In nature, *Bacillus anthracis* exist in the form of spores that are metabolically indifferent and resistant to environmental conditions. Spores are highly resistant to heat, ultraviolet, ionizing radiation, extreme pressure, and chemical disinfectants [44,45,46]. Anthrax is usually a disease of herbivores that ingest or inhale spores while grazing (cattle, sheep, and goats). Disease in humans can occur through contact with infected animals or animal products. Such cases took place in northeastern Turkey in 2018. The residents of a village showed symptoms of anthrax after consumption of infected beef. Anthrax can be contracted by humans in four different ways: by inhalation, ingestion, through the skin, and by injection [44,47]. The pathogenesis of *Bacillus anthracis* is mainly caused by two virulence factors: tripartite exotoxin and a poly-γ-D glutamic acid capsule (γ-DPGA). Plasmid pXO1 includes genes for components of exotoxin: lethal factor (LF), edema factor (EF), and protective antigen (PA). These proteins are individually non-toxic, but together, as edema toxin (PA + EF) and lethal toxin (PA + LF), cause detrimental effects. In the meantime, plasmid pXO2 includes genes for the synthesis of the capsule, which prevents bacterial lysis and phagocytosis by cationic serum proteins [48]. All of these elements are necessary to complete virulence action. Protective antigen (PA) is the major virulence factor of Bacillus anthracis, which binds itself to the host cell surface receptor and mediates endocytosis of lethal factor (LF) and edema factor (EF) into the cells [49]. Two types of receptors on target cells can bind PA: tumor endothelial marker 8 (TEM8 or ANTXR1) expressed by epithelial cells of the skin, lung, or intestine, and capillary morphogenesis protein 2 (CMG2 or ANTXR2), expressed by other types of human tissue. The affinity of PA for CMG2 was shown to be higher than for TEM8, by 11-fold in cell culture and 1000-fold in vitro. Upon binding to the receptor at the cell surface, the 83 kDa form of PA (PA_83_) is cleaved by a furin-like protease and leading to the receptor-associated PA_63_ and release of PA_20._ In response to PA binding, receptors are phosphorylated on tyrosine residues by Src or Fyn kinases and ubiquitinated by Cbl (for TEM8 receptor) or an unknown E3 ligase (for CMG2 receptor). After oligomerization in lipid rafts, toxin-receptor complex is internalized from the cell surface by endocytosis. Next, PA undergoes conformational change and forms pores. The anthrax toxin complexes transit from the early to late endosomal multivesicular compartments. The endosomal acidification leads to conversion of the PA oligomer structure pre-pore to a protein-conducting channel, through which EF and LF are transported into the cytosol to exert their cytotoxic results. LF and EF can also be released into the cytosol through back fusion of the intraluminal vesicles with endosome membranes [50,51]. LF is a zinc-dependent metalloprotease that cleaves and inactivates members of the mitogen-activated protein kinase kinase (MAPKK) protein kinase family. Downstream of MAPKKs are the extracellular signal-regulated kinase (ERK), c-Jun N-terminal kinase (JNK), and p38 signaling pathways involved in various cellular processes, including growth, response to diverse form of cellular stress, cell fate determination, and apoptosis. EF is a highly efficient, calmodulin-dependent adenylyl cyclase, which shares homology with adenylate cyclase toxins ExoY from *Pseudomonas aeruginosa* and CyaA from *Bordetella pertussis*. EF can convert up to 2000 molecules of ATP to cAMP per second, significantly increasing the concentration of this classical second messenger, thereby causing diverse effects [52]. There are three forms of anthrax (inhalational, gastrointestinal, and cutaneous) depending on the route of exposure to *B. anthracis* [53]. Inhalational anthrax is considered to be the most fatal form of anthrax. Without treatment, the fatality rate is nearly 95%, while immediate intervention can lower the fatality rate to 50%. Macrophages play a central role in the initiation of inhalation anthrax infection. When spores are deposited in the lungs, they are engulfed by alveolar macrophages, where spores germinate and become vegetative bacteria. Macrophages transport spores to the regional tracheobronchial lymph nodes [54,55]. Then, vegetative cells become encapsulated, and destroy phagosome and cellular membranes for the escape from the macrophage. In the regional lymph nodes, the production of virulence factors by proliferating bacteria causes the formation of a characteristic pathological picture of mediastinitis, edema, massive hemorrhage, and tissue necrosis in the lymph nodes. When the clearing ability of the regional nodes is overwhelmed, lymph nodes are damaged, opening access into the circulation to nodes blood vessels and bacteria, which can spread to the blood causing sepsis [56]. Gastrointestinal anthrax occurs as a result of consumption of improperly prepared meat or meat products from animal infected with anthrax. The mortality rate is variable and can reach ≤40% with appropriate antibiotic treatment [46]. Gastrointestinal anthrax is present in two clinical forms: intestinal and oropharyngeal. The spores enter the mucosal and submucosal lymphatics through breaches in the mucosal lining of the intestinal tract or through phagocytic and antigen-processing cells, for example, M cells. Macrophages transport spores to mesenteric and other regional lymph nodes, where spores germinate, multiply and attack other tissues indirectly through the blood or directly by peritoneal cavity. In oropharyngeal form, spores are acquired through ingestion and inhalation and spread to the cervical lymph nodes. If the infection generalizes, its development can be compared to systemic inhalational anthrax [56,57]. Oropharyngeal form is characterized by sore throat, enlargement of cervical lymph nodes, mucosal ulcerations, and soft tissue edema. Guts ulceration, generally in the region of the cecum and ileum, is a clinical symptom of intestinal form. Additional manifestations include anorexia, nausea, vomiting, abdominal pain, melanotic stool, and bloody diarrhea [46]. Cutaneous anthrax normally occurs through skin contact with infected animals or animal products. This form of anthrax composes 95% of human cases globally. Fatality rate is <1% with the treatment. The duration of incubation varies between 3 and 7 days. The clinical picture ranges from mild to severe form. Mild cutaneous anthrax is characterized by presence of a cutaneous lesion with <4cm diameter, which is surrounded by erythema, but without systemic symptoms. Severe form of anthrax is defined by the presence of a large cutaneous lesion, which is surrounded by a ring of blisters with serous fluid and an area of edema that may become extensive. The lesion is often described as a “malignant pustule” due to its distinctive look. Coagulation necrosis is the cause of its dark color. Systemic symptoms contain fever, tachycardia, hypotension, regional lymphadenopathy, and tachypnea. In some cases, complications, such as sepsis, toxemic shock, and other organ involvement may occur [47,56,58]. Antibiotic therapy is used in the treatment of anthrax infection. Penicillin, doxycycline, ciprofloxacin. Antibiotic therapy must begin soon after exposure, as its effectiveness reduces as toxemia progresses. Anthrax vaccines are also important in the treatment. This combination therapy assumes that germinated spores will be killed by antibiotic therapy, but later infection resulting from latent spore germination, which may appear after completion of antibiotic therapy, will be prevented by immune response induced by vaccination. The Centers for Disease Control and Prevention (CDC), Advisory Committee in Immunization Practices (ACIP) recommend vaccination with Anthrax Vaccine Absorbed (AVA), and, additionally, 60-day antibiotic therapy in case of confirmed or suspected exposure to anthrax spores [58,59,60]. The only licensed anthrax vaccine, Anthrax Vaccine Absorbed (AVA, BioThrax^®^), is advisable for active immunization in humans 18–65 years of age at high risk of exposure. The primary immunogen in AVA is PA. AVA induces immunity primarily by stimulating the production of antibodies against PA. Anti-PA antibodies directly neutralize the toxin, inhibiting spore germination, and improve the spore phagocytosis by macrophages [60,61]. 

### 2.2. Yersinia pestis

*Yersinia pestis* is a small, non-motile, Gram-negative bacterium, which belongs to the Enterobacteriaceae family. *Y. pestis* is the causative agent of plague, a rare but highly lethal zoonosis [62]. Fleas are important vectors in plague transmission to small mammal species, especially rodents, which indicate natural reservoirs in environment [63]. Most human infections result from contact with infected animals, or from the bite of an infected flea [64]. Plague is endemic in more than 25 countries worldwide. Despite effective antibiotic therapy, mortality in endemic regions is greater than 10%, mainly as a result of the fast development of pathogenesis [65]. *Yersinia pestis* determinants are secreted through a type three secretion system (T3SS). 

The T3SS is encoded in the *Yersinia* virulence plasmid pYV/pCD1. The T3SS’s function is to inject multiple toxic *Yersinia* effector proteins (Yops) directly into the eukaryotic host cell cytosol and refute cell signaling pathways, leading to apoptosis. Yops are also responsible for inhibition of phagocytosis and downregulate the production of proinflammatory cytokines. Apart from the T3SS virulence plasmid, there are two other plasmids (pPla/pPCP1, pFRa/pMT1) that are unique to *Yersinia pestis* virulence. The protease Pla is encoded in pPla/pPCP1 plasmid and its outer membrane protein is characterized by adhesive, coagulase, and fibrinolytic properties. Pla converts plasminogen to plasmin and, as a result, degrades extracellular matrices, giving bacteria the ability to rapidly invade the host cell and migrate to lymph nodes. The pFRa/pMT1 plasmid is responsible for the production of a murine toxin, which is needed during the colonization of fleas [31,66]. Plague mainly occurs in three forms: pneumonic, bubonic, and septicemic plague [67]. The most severe manifestation of plague, and the most rapidly developing, is the pneumonic plague, where the mortality rate approaches 100% in the absence of treatment. The primary pneumonic plague is caused by aerosol exposure to *Yersinia pestis*, resulting from the inhalation of infectious aerosol droplets. Secondary pneumonic plague develops from dissemination of bacteria into the lungs during septicemic and bubonic forms of disease. The pneumonic plague is transmitted from person-to-person through respiratory droplets. After an incubation period of 2–4 days, various symptoms of disease include fever, headache, nausea, malaise, vomiting, productive cough with bloody sputum, difficulties breathing, and chest pain. If appropriate antibiotic therapy is administered within 24 h after the symptoms appear, mortality rate can be lowered by up to 50%. Without treatment, mortality rates approach 100% [31,64,68]. The bubonic form is the most common, resulting from the bite of an infected flea. Bubonic plague is characterized by the formation of buboes (swollen lymph nodes) [69]. Usual incubation period varies from 2 to 6 days, occasionally longer. During the incubation, *Y. pestis* disseminates from the dermis to regional lymph nodes. Bacteria avoid immune clearance and proliferate. The first symptoms include fever, headache, muscle pain, and arthralgias. This form usually develops regional red, dry, and hot skin, and swollen lymph nodes, which appear in the armpits, groin, neck, or site of the insect bite. Infected lymph nodes become severely damaged by hemorrhage and necrosis. Bubonic plague is the dominant form of plague and accounts for 80–95% of cases. Fatality rate is 10–20% [12,68]. If bubonic plague is not diagnosed and treated at the appropriate time, it can develop into septicemic plague, by spreading bacteria via blood. This form of plague can also result from direct entry of *Yersinia pestis* through damaged skin or mucous membrane by the infective flea bite. The usual incubation period is 2–7 days, but this type of plague can lead to death before clinical manifestations even appear. Symptoms of septicemic plague include abdominal pain, bleeding into the skin, and other organs. Skin and other tissues may become necrotic, especially the nose, fingers, and toes. Moreover, fever, diarrhea, vomiting, and weakness can be observed. Septicemic plague stands out by high bacteremia and is accompanied by a dangerous endotoxemia [68,70]. The successful treatment of plague is early recognition, administration of efficient antibiotics, and anti-shock therapies. If the therapy is late by more than 24 h after infection, it could be fatal for patients. Most of *Yersinia pestis* isolates are sensitive to streptomycin, however, other antibiotics, such as gentamycin, doxycycline, and fluoroquinolones are considered as alternatives. Furthermore, a multidrug resistant (MDR) strain was isolated in Madagascar. In addition to antibiotic therapy, treatment for shock should not be ignored [12,71]. Currently, there is no available licensed plague vaccine, despite plenty of studies having been conducted for many years worldwide. Derbise et al. showed that subcutaneous vaccination with the recombinant attenuated *Yersinia pseudotuberculosis* vaccine strain VTNF1 successfully immunized and protected mice against pneumonic and bubonic plague. Furthermore, the optimal dose was lower than needed for oral vaccination [65]. A study by Chen et al. showed that the *Yersinia pestis* F1-liposome vaccine, using microneedles as a delivery system, was efficient in mice [63]. At present, plagues occur in Asia, Africa, and the Americas. Outbreaks in China, Uganda, and Democratic Republic of Congo are showing that plague is still a serious public health threat. In September 2017, the Madagascar Ministry of Public Health reported to the World Health Organization (WHO) an outbreak of pneumonic plague, where a total of 2348 confirmed and suspected cases occurred, including 202 deaths, causing a regional panic [31,70]. 

### 2.3. Francisella tularensis

*Francisella tularensis* is a Gram-negative, non-motile, non-sporulating coccobacillus. It is a small, intracellular pathogen that is characterized by high virulence and a low infective dose (1–10 cells) [72]. At the moment, four subspecies of *F. tularensis* have been identified: *tularensis*, *holarctica*, *novicida,* and *mediasiatica*. *Francisella tularensis* ssp. *tularensis* is the most virulent subspecies and occurs in North America. The *holarctica* subspecies usually causes milder forms of disease and can be found in Asia and Europe. *Francisella tularensis* ssp. *novicida* and *mediasiatica* rarely cause diseases in humans. *F. tularensis* causes a disease called tularemia in humans and animals. The main transmission vectors are arthropods (deerflies, ticks), when small mammals (rabbits, hares, squirrels, water rats) are reservoir hosts [73,74]. In general, terrestrial and aquatic cycles for *F. tularensis* can be distinguished. Terrestrial cycle is related to *Francisella tularensis* ssp. *tularensis*. Lagomorphs and rodents are reservoirs and ticks are the main vector. Aquatic cycle is associated with *Francisella tularensis* ssp. *holarctica* and semi-aquatic or small mammals living near water are reservoirs, with mosquitoes being the vector. Bacteria can be further transmitted through contact with contaminated water. Inhalation of infected aerosol, or direct contact with tissues and liquids of infected animals, can also be used as a route for bacterial transmission [75]. Transmission by direct contact is possible while skinning, therefore, hunters and forest workers are at an increased risk of infection [76]. *Francisella tularensis* is capable of infection and replication within different host cells (macrophages, dendritic cells, neutrophils, or hepatocytes). Thus, bacteria are able to circumvent host defense systems and receive entry to the cytosolic environment [77]. Bacteria enter the host macrophages via unique mechanisms, whereby *F. tularensis* is engulfed through asymmetrical pseudopod loops of the macrophage. The lipopolysaccharide (LPS) of bacteria, somewhat different from classical endotoxin in that it induces low levels of proinflammatory cytokines, mediates this process. Interaction with the macrophage complement receptor blocks the oxidative burst and the activation process of the macrophage. Bacteria have the ability to replicate inside the host macrophages, using a large amount of virulence genes, e.g., mglA and mglB (macrophage growth locus), which resemble a Type IV secretion system, similar to that of *Pseudomonas aeruginosa* and *Vibrio cholerae*. In the phagosome compartment of the macrophages, *F. tularensis* eludes phagolysosome fusion, which exits phagosome and replicates inside the cytosol. Within the cytosol of an infected cell, bacteria induce a unique form of apoptosis and pyroptosis, and results in the release of *F. tularensis* from the cell [78,79]. There are six major forms of tularemia, classified on the basis of symptoms: pulmonary, glandular, ulceroglandular, oropharyngeal, typhoidal, and oculoglandular [76]. The incubation period of the disease is usually 3–5 days after exposure. Ulceroglandular form is the most common, and is a result of a bite of the arthropod vector or an infection acquired through skin during contact with an infected animal. After the incubation period, this form is characterized by fever and ulcers with a necrotic base, and a sharply punched-out border. Tender regional lymphadenopathy develops within a few days of onset [80]. Glandular form of tularemia may occur after the entry of bacteria into the organism through abrasion. Tender regional lymphadenopathy, without evident cutaneous lesions, are observed. Oropharyngeal form may develop after consumption of undercooked, infected food, or contaminated water, after which the bacteria inoculates the pharynx instead of the skin. Patients often present fever, throat pain, cervical lymphadenopathy with infra auricular nodal involvement. Pharyngeal ulcers and exudative or membranous pharyngotonsillitis may appear. Abdominal pain, diarrhea, and emesis are also observed. Furthermore, in a severe form of the disease, gastrointestinal bleeding as a result of intestinal ulceration may be present. The oculoglandular tularemia is pathologically similar to the ulceroglandular form, but the conjunctival sac is its primary place of inoculation. Clinical manifestations, such as conjunctival ulcers, unilateral purulent conjunctivitis and periorbital edema, are typical. Corneal ulceration and perforation can be a possible complication of the oculoglandular form of disease [81,82]. The respiratory route of infection is the most severe one, which causes a pneumonic form of tularemia [83]. A primary pneumonic form of the disease typically occurs by inhalation of aerosolized bacteria directly into the lungs from infected animals or soil, water, and hay contaminated by infected animals [84]. Secondary pneumonic tularemia appears when bacteria enter the circulation system and spread to the lungs from another site of infection. Secondary pneumonic form can occur with any other form of the disease. Symptoms include high fever, nonproductive cough, chest pain, and hilar adenopathy; pulmonary infiltrates or pleural effusions may also appear [85,86]. Primary symptoms of typhoidal form are fever, chills, and severe fatigue. Then, vomiting, diarrhea, delirium, and abdominal pain occur. Clinical manifestations also include general fatigue, sepsis, and death. Typhoidal form is the most difficult to diagnose due to its general symptoms being without an obvious external lesion or regional lymph node swelling [81,87]. Early diagnosis and initiation of appropriate antibiotics are essential. Administration of aminoglycosides (streptomycin, gentamycin) for 7–14 days is recommended as a drug of choice for the severe form of the disease. However, tetracycline, chloramphenicol, and quinolones are also efficient [80,88]. Currently, there is no vaccine approved for the prevention of tularemia. However, studies into live-attenuated vaccines have provided promising results, simultaneously revealing a significant impact of T cell-mediated events in the control of *Francisella tularensis* [13].

### 2.4. Staphylococcus aureus

*Staphylococcus aureus* is an aerobic, Gram-positive bacteria, which commonly colonizes the nasal cavity and skin of healthy people. The prevalent use of antibiotics, especially in the medical sector, has resulted in increased bacterial resistance to antibiotics, the example of which is methicillin-resistant *Staphylococcus aureus* (MRSA). This organism is resistant to all β-lactam antibiotics, including nafcillin, oxacillin, methicillin, and cephalosporins [89,90]. The occurrence of MRSA is the result of the most likely inappropriate use of a broad spectrum of β-lactam antibiotics. *Staphylococcus aureus* resistant to methicillin is a significant public health problem, especially in hospitals, which are reservoirs of pathogens and play an important role in their transmission. Moreover, MRSA is one of the most common bacterial illnesses in hospitals. MRSA has been detected in the majority of countries around the world, but there are differences in scale of prevalence [91,92]. MRSA strains are divided into two types: community-acquired MRSA (CA-MRSA), isolated from outpatients, and hospital-acquired MRSA (HA-MRSA), isolated from hospitalized patients. CA-MRSA strains are susceptible to non-β-lactam antimicrobial agents, but produce different virulence factors, such as a Panton–Valentine leucocidin, exfoliative toxin, or arginine catabolic mobile element. Unlike CA-MRSA, HA-MRSA strains show high resistance to a variety of non-β-lactam antibiotics, such as macrolides, quinolones, and aminoglycosides. *S. aureus* develops β-lactam antibiotic resistance by obtaining the gene *mecA*, which is located on the mobile genetic element, called staphylococcal cassette chromosome *mec* (SSC*mec*). This element shows high diversity in genetic content and structural organization and can be classified into many types, subtypes, and variants [93,94]. SSC*mec* consists of three major structural elements: the *mec* gene complex, the cassette chromosome recombinase (*ccr*) gene complex, and the joining (J) regions. The *mec* gene complex includes the *mec*A gene, which determines the resistance of staphylococci to methicillin, and is responsible for penicillin binding protein 2a (PBP2a) production. PBP2a has a reduced affinity for β-lactam antibiotics. In *mec* gene complex, there are also *mec*I and *mec*R1 regulator genes, encoding respectively a signal repressor protein and transducer protein, as well as different insertion sequences [95,96]. The *ccr* gene complex consists of *ccr*A, *ccr*B, and *ccr*C genes. This complex encodes recombinases, which are responsible for the excision and specific integration of the SCC*mec* into the *Staphylococcus* bacteria chromosome. The *ccr* gene complex also ensures the SCC*mec* mobility and allows its transfer to another *Staphylococcus* species. Joining regions can be divided into three associated regions: J1, J2, and J3, which may contain non-coding sequences, pseudogenes, plasmids, and transposons [97]. It is estimated that in the USA, 42.6–44% of surgical site infections caused by *Staphylococcus aureus* are MRSA [98]. The main transmission route of *S. aureus* resistant to methicillin is direct contact between patients and health care personnel. Substantial MRSA colonization has been reported on hospital linens and health care workers’ clothes after their contact with MRSA carriers [99,100]. MRSA infection leads to skin infections, pneumonia, bloodstream infections, sepsis, surgical site infections, and, in severe cases, death [101]. The WHO report informs that patients infected with MRSA are 64% more likely to die than patients infected with a non-resistant bacteria [99]. The development of new medicines and strategies in treatment of infections with MRSA are the main public health issues. Greber et al. have shown in their research that lipopeptide compounds, such as (C_10_)_2_-KKKK-NH_2_ and (C_12_)_2_-KKKK-NH_2_ could be an alternative to daptomycin, which is applied in bacteremia, skin and soft tissue infections caused by MRSA [102]. Furthermore, hand hygiene and antimicrobial surface cleaning are important. These types of actions effectively reduce the exposure of patients to HA-MRSA [101].

## 3. Viral Pathogens with High Impact Potential on Public Health

### 3.1. Ebola Viruses 

Ebola viruses (EBOVs) belong to the *Filoviridae* virus family and are characterized by a linear, non-segmented, negative stranded RNA structure [103]. *Filoviridae* virus family includes one of the genera, Filovirus, which comprises two morphologically identical, but serological separate species: Ebola virus and Marburg virus. There are five subtypes of Ebola named after the region of their discovery: Ebola virus (ZEBOV), Sudan ebolavirus (SEBOV), Bundibugyo ebolavirus (BDBV), Reston ebolavirus (REBOV), and Tai Forest ebolavirus (TAFV). Zaire, Sudan, and Bundibugyo EBOVs are responsible for the large outbreaks in Africa. REBOV and TAFV were not accounted for within the illness or mortality rates in humans [104,105]. The specific host reservoir for EBOV remains unknown. The African fruit bat seems to be the natural reservoir for this virus and bats can transfer the virus to monkeys, forest antelopes, or porcupines. Ebola can be introduced into the human organism through the contact with meat, blood, or secretions of infected animals [103]. The structure of enveloped EBOV virion consists of a nucleocapsid complex, surrounding matrix and coating envelope. The nucleocapsid complex is created by viral RNA genome packed with nucleoprotein (NP), polymerase (L), viral proteins VP35 and VP30. NP has two functions: protection of the viral RNA against exogenous nucleases and encapsulation of the genome during virus assembly. VP35 plays role in dissociation of NP-RNA complexes. Moreover, it inhibits production of type I interferon and dendritic cell maturation by interfering with host retinoic-acid-inducible gene I-like receptor (RLR) signaling [106,107]. VP30 and L play a key role in the activation of viral RNA synthesis. VP40, the matrix protein, maintains the structural integrity of the virion and directs budding of viral particles from the cell surface. VP24 is a minor matrix protein, which plays various roles in virus release, nucleocapsid assembly, and immune response suppression. Glycoprotein (GP) is essential in viral pathogenesis. This is the only surface protein present, as trimeric spikes comprise GP1 and GP2, and can mediate attachment and entry into the host cell by receptor binding and fusion. GP1 mediates the binding between virus and target cell, and GP2 function is membrane fusion [45,106]. EBOV virulence is imputed to the ability of the virus to avoid innate antiviral responses by interfering with or disabling and signaling functions of immune cells, such as macrophages and dendritic cells. EBOV can also suppress dendritic cells maturation and the effect is unsettled development of effective adaptive immune response. It can lead to systemic viral replication. In vivo studies in non-human primate models have showed that EBOVs substantially infect adrenal cells, endothelial cells, monocytes, and Kupffer cells in the liver. After membrane fusion, NP is released into the cytoplasm where viral RNA is translated into mRNA by host cell machinery. Then, L, GP, VP40, VP35, and VP24 are translated from viral mRNA by the host machinery. Subsequently, NP, L, and VP35 act like viral polymerase complexes and admit viral genome replication. Synthesized proteins undergo various post-translational modifications and accumulate in the lipid raft along with the encapsulated viral genome on the cell membrane. Then, interaction of VP40 with the host endosomal sorting complexes required for transport (ESCRT) allows viral budding and delivery of a new virion [103,108].

Ebola virus disease (EVD) also known as Ebola hemorrhagic fever is caused by Ebola virus. EVD spreads human to human and its estimated mortality rate ranges between 40 and 90% depending on the conditions, epidemics, and access to care. The major mode of transmission of EBOV is personal contact with blood, vomit, mucus, feces, or other bodily fluids. The largest outbreak occurred in West African countries and then spread into Spain, USA, Italy, and UK via transport of patients and individual case exportation. From December 2013 to April 2016, in Guinea, Liberia, and Sierra Leone, a total of 28,616 confirmed and suspect cases were reported. During this outbreak, 11,310 deaths, according to the World Health Organization (WHO), were reported [20,109]. An incubation period can range from 2 to 21 days. Clinical descriptions enumerate non-specific symptoms of EVD such as fever, asthenia, myalgia, vomiting, diarrhea, conjunctivitis, delirium and dyspnea [110]. These early symptoms can be similar to other diseases, such as malaria, Lassa fever, typhoid, borreliosis, shigellosis, rickettsia diseases, or viral hepatitis [105]. Next, vascular dysfunctions occur as petechiae, ecchymoses, mucosal bleeding, bleeding at needle puncture sites, or diffuse coagulopathy. Then, gastroenteritis with dehydration, multiorgan failure (kidneys, liver, respiratory, and coagulation system), and sepsis may appear. Death is preceded by the appearance of hypotension, tachycardia, tachypnea, and anuria. Levels of viremia in the infected patient’s blood has been correlated with the lethality of the disease [105,111,112]. Currently, numerous treatments for EVD have entered the clinical trial phase. Presently, there is only one Food and Drug Administration (FDA) approved vaccine for Ebola virus’s prevention. This is the rVSV-vectored vaccine which showed 100% efficacy [113]. Supportive medical care should be initiated. The virus reproduces and spreads in the organism, disturbs electrolyte balance, and interferes with blood clotting. The consequence of this is dehydration and patients need intravenous fluids and oral rehydration. Moreover, broad spectrum of antibiotics, antiemetic drugs, and nonsteroidal anti-inflammatory medications can be used. Nutritional support is also important. Furthermore, treatment with renal replacement therapy, mechanical ventilation, and oxygen therapy can be effective to prevent the patient’s health from deteriorating, and death [103,114,115]. 

### 3.2. Influenza Viruses

Influenza viruses are among the most common causes of respiratory tract infections in humans and the most important because they cause high morbidity [116]. Influenza viruses are member of *Orthomyxoviridae* family of viruses and are negative strand RNA viruses. There are four types of influenza viruses: A (IAV), B (IBV), C (ICV), and D (IDV). Influenza A and B viruses are responsible for causing influenza in humans [117]. IAV has been identified in seasonal epidemics in the winter months and global pandemics. IBV is associated with seasonal epidemics, but are not typically with pandemic [118]. All influenza viruses are enveloped, negative-sense single-strand RNA viruses. Influenza A and B viruses contain eight genomic segments, which encode which encode transcripts for 10 essential viral proteins, as well as several strain-dependent accessory proteins, such as hemagglutinin (HA), neuraminidase (NA), polymerase basic 2 (PB2), polymerase basic 1 (PB1), polymerase acidic (PA), nucleoprotein (NP), matrix protein (M1), membrane protein (M2), non-structural (NS1), and nuclear export protein (NEP) [119]. The HA and NA proteins are the most antigenically variable, and in the case of influenza A virus, they are divided into antigenically diverse subtypes. They are located at the surface of the virus particle and are the main targets for protective antibodies induced by influenza virus infection or vaccination. Influenza C and D viruses have seven RNA segments and do not appear to cause significant disease in humans. However, influenza C infection can cause flu-like illness in some cases, especially in children [120,121]. A unique feature of IAVs is that they circulate not only in humans but also in domestic animals, wild aquatic birds, poultry, pigs, and horses. The ability to adapt to multiple species is a main cause why IAVs are more diverse than IBVs, which are essentially exclusive to humans. A total of 9 antigenically different NA and 16 antigenically different HA serotypes or subtypes have been identified amongst the different avian strains of IAV [122]. Despite their antigenic differences, the viral proteins encoded by the influenza A and B virus genomes have similar functions. Three RNA segments encode three subunits of the viral RNA-dependent RNA polymerases (PB1, PB2, and PA), which are responsible for RNA synthesis and replication in the infected cells. Two RNA segments encode the viral glycoproteins: HA which mediates binding to sialic acid-containing receptors and viral entry, and NA that is accountable for releasing viruses bound to non-functional receptors and helping in viral spread. RNA segment 5 encodes NP and the RNA genome is bound by this protein. RNA segments 6 and 8 encode more than one protein, viz. M1, M2 (BM2 in the case of influenza B), NS1, and NEP. The M1 protein provides a scaffold which supports the structure of the virion and together with NEP regulates the trafficking of the viral RNA segments in the cells. The M2 protein is a proton ion channel which is required for viral entry and exit. Together with the HA and NA glycoproteins, is situated on the surface of the virus anchored in a lipid membrane from the infected cell. The NS1 protein is a virulence factor, which inhibits host antiviral responses in infected cells [121,123]. The IAV attacks host cells through binding to receptors containing sialic acid. The human IAVs prefer α-2,6-linked sialic acid, while the avian influenza viruses bind to α-2,3-linked sialic acid (SA). Swine cells contain two receptor types: α-2,6 type–binding SA (they appear in human respiratory cells) and the α-2,3 type (found in birds) [124,125]. Once viral molecules have bound to the receptors, they migrate to endosome and the viral ribonucleoprotein (RNP) complex is released into cytoplasm. The released RNP complex is next transported to the cell nucleus where viral transcription and RNA replication occur [124,126]. The virus is transmitted from person to person mainly via aerosol during coughing or sneezing. It occurs when the droplets produced by ill people reach by air the oral or nasal mucosa of a healthy person nearby. Moreover, it may happen when healthy persons touch objects covered with the droplets produced by an ill person and then, before washing hands, pass them on to their (or others’) oral or nasal mucosa [127]. The influenza syndrome is sudden in onset and is characterized by headache, fever, cough, sore throat, rhinorrhea, nasal congestion, myalgia, weakness, and loss of appetite [128]. Additionally, gastrointestinal symptoms including abdominal pain, nausea, vomiting, and diarrhea are common [129]. Influenza infection can cause extra-pulmonary complications. Majority of extra-pulmonary complications are related with the acute phase of the infection and frequently manifest as the presenting symptoms. Data suggest that extra-pulmonary complications, including cardiac (cardiac ischemia, cardiomyopathy, arrhythmia), neurologic (cerebrovascular accident, Guillain–Barre syndrome, encephalitis), renal (acute kidney injury, acute tubular necrosis), or hepatic (hepatitis, hepatic vein thrombus) can occur [130]. Influenza occurs in all age groups and cause yearly seasonal epidemics with their peaks in the countries of a temperate climate, especially during autumn and winter. In some tropical countries, influenza occurs throughout the whole year and worsens in the rainy season [131]. Influenza is usually self- limiting in healthy people. Treatment of uncomplicated disease is supportive and usually includes antipyretics, sufficient fluid intake, rest, and staying at home to limit spread to others [131]. Vaccination is seen as the best option to prevent, control, and decrease the socioeconomic burden of influenza. Vaccination can also reduce illness and lessen severity of infection. However, the current seasonal vaccines require annual evaluation and reformulation [132]. A major challenge in combating IAV is the continuous evolution of surface antigens (HA and NA) in response to pressure from the host’s immune system, which is termed antigen drift and antigenic shift [133]. Antigenic drift is the progressive accumulation of point mutations on the influenza virus’ surface glycoproteins HA and NA, driven by high error rates of the virus’ RNA-dependent RNA polymerase. Moreover, the mutations are positively selected and become fixed, resulting in new strains that differ antigenically from what the host was vaccinated against [134]. The antigenic shift is responsible for the development of the pandemic, and is the reassortment process of gene segments across various strains infecting the same host, resulting in a complete change in antigenicity [122].

### 3.3. SARS-CoV-2

Severe acute respiratory syndrome coronavirus 2 (SARS-CoV-2) is the new human beta-coronavirus after the previously identified SARS-CoV and the Middle East Respiratory Syndrome coronavirus (MERS-CoV). SARS-CoV-2 is responsible for the 2019 coronavirus disease (COVID-19), which is an ongoing worldwide pandemic [135]. SARS-CoV-2 is an enveloped, non-segmented, positive sense RNA virus, which is broadly distributed in humans and other mammals. A samples isolation from pneumonia patients found that strains of SARS-CoV-2 had a length of 29.9 kb [136]. Gene fragments express structural and nonstructural proteins. The spike S, E, M, and N genes encode structural proteins, while nonstructural proteins, such as papain-like protease, 3-chymotrypsin-like protease, and RNA-dependent RNA polymerase are encoded by the open reading frame (ORF) region [137]. Structural proteins, including the spike (S), envelope (E), membrane (M), and nucleocapsid (N) proteins have high sequence similarity to the sequence of the corresponding SARS-CoV and MERS-CoV proteins [138]. A large number of S glycoproteins cover the surface of the virus and bind to the angiotensin converting enzyme 2 (ACE2) receptor in the host cells. S glycoprotein includes two subunits, S1 and S2. S1 subunit determines the virus-host range and cellular tropism with the receptor binding domain (RBD), whereas S2 mediates virus-cell membrane fusion [139,140]. The S protein is cleaved by a host cell protease, the transmembrane protease/serine subfamily member 2 (TMPRSS2), an airway and alveolar cell serine protease expressed on epithelial cells of the respiratory tract, like type II pneumocytes. TMPRSS2-mediated cleavage and priming of S glycoprotein is required for binding to ACE2, membrane fusion and cell entry [141]. The nucleocapsid (N) protein is the structural component of CoV localizing in the endoplasmic reticulum-Golgi region that structurally is bound to the nucleic acid material of the virus. Its function is to enter the host cell, bind to the viral RNA genome and form the ribonucleoprotein core [136,142]. The E protein is the smallest of the main structural proteins. During the replication cycle, E protein is profusely expressed inside the infected cell, but only a small part is incorporated into the virion envelope. The most of the protein is situated at the site of intracellular trafficking, namely Golgi, endoplasmic reticulum (ER) and endoplasmic-reticulum–Golgi intermediate compartment (ERGIC), where it is involved in the virus assembly and budding. Studies with recombinant coronavirus (CoV) lacking E showed significantly reduced viral titers or impaired viral maturation, indicating the importance of protein E in virus production and maturation [143,144]. The M protein is the most abundant structural protein and determines the shape of the virus envelope. Moreover, M protein can interact with all other major coronaviral structural proteins [136]. Interaction with S protein is necessary for retention of S protein in the ERGIC/Golgi complex and its incorporation into new virions, but dispensable for the assembly process. Binding of M to N protein stabilizes the nucleocapsid (N protein-RNA complex) and internal core of virions, as well as promotes completion of viral assembly. Together, M and E proteins form a viral envelope, and their interaction is sufficient to generate and release virus-like particles (VLPs) [144,145]. Zhou et al. have demonstrated, that the SARS-CoV-2 uses the same cell entry receptor, ACE2, as SARS-CoV [146]. For cellular entry, the S glycoprotein binds to ACE2 and is primed by TMPRSS2, that promotes endocytic entry of the virus [147]. Once the CoV is inside the host cell via membrane fusion, the viral genome RNA is released into the cytoplasm, where the uncoated RNA translates two large polyproteins (pp1a and pp1ab), that encode non-structural proteins and also form replication-transcription complex (RTC). The RTC continuously replicates and synthesizes a nested set of subgenomic RNAs that encode structural and accessory proteins. Mediating Golgi and ER, newly formed genomic RNA, envelope glycoproteins and nucleocapsid proteins assemble and form viral particle buds. Finally, virion containing vesicles bind to the plasma membrane, releasing the virus [148,149]. Viral entry and cell infection trigger the host’s immune response. The inflammatory cascade is initiated by antigen-presenting cells (APC), which present the foreign antigen to CD4 + -T (Th1) helper cells and release interleukin-12 (IL-12) for further stimulation of the Th1 cells. The Th1 cells stimulate CD8 + -T-killer (Tk) cells which target all cells containing the foreign antigen. Moreover, activated Th1 cells stimulate B-cells to produce antigen-specific antibodies [150]. The pulmonary system is the most commonly affected organ system by COVID-19. The most frequent clinical manifestations include fever, fatigue and dry cough [151]. In the severe disease, pneumonia with acute respiratory distress syndrome (ARDS), acute hypoxic respiratory failure and death can occur [152]. While the pulmonary system is most commonly affected, COVID-19 can affects extrapulmonary systems (including renal, gastrointestinal, cardiovascular or neurologic systems), which can have serious health implications. SARS-CoV-2 can cause both immediate cardiovascular and indirect cardiovascular outcomes, including acute coronary syndromes (ACS), myocardial injury, cardiomyopathy, arrhythmias, cardiogenic shock, or thrombotic complications [153]. Analyzes indicate that acute kidney injury (AKI) is a common complication of COVID-19 and is associated with mortality. The most prominent risk factors for the development of AKI were severe COVID-19, in particular the need to support ventilation or treatment with vasopressors [154,155]. Other research suggests that gastrointestinal symptoms and liver damage are not uncommon in COVID-19 patients. Patients with severe COVID-19 had a higher risk of gastrointestinal symptoms and liver damage. The most common symptoms were lack of appetite, diarrhea, vomiting, and abdominal pain [156,157]. As in the previously described cases, patients with severe COVID-19 were more likely to develop neurological symptoms, especially impaired consciousness, acute cerebrovascular disease and skeletal muscle injuries. Most of the neurological symptoms occurred in the early stages of the disease [158]. SARS-CoV-2 is highly transmissible from person to person via respiratory droplets and secretions. At present, therapeutic strategies to deal with the infection are only supportive, while isolation, reduced contact with other people and good hygiene practice are effective prophylaxis to limit community transmission [25,159]. No specific antiviral drug has been shown to be effective in treatment of patients with severe COVID-2019. Beigel and colleagues conducted a placebo-controlled trial of an antiviral drug, remdesivir, in adult patients with COVID-19, and had symptoms of a lower respiratory tract infection. The data showed that the recovery time in patients who received remdesivir compared to patients who received placebo was shorter. The results also show that treatment with remdesivir can prevent progression to more severe respiratory disease. This reduces the possibility of side effects caused by respiratory failure and a lower frequency of respiratory support [26]. A study in patients with severe COVID-19 showed that all patients with convalescence plasma (CP) transfusion obtained a negative serum SARS-CoV-2 RNA result. In addition, increase of lymphocyte counts, oxygen saturation and the improvement of liver function and C-reactive protein (CRP) occurred. The results suggest that CP therapy could be an easily available and safe treatment option for severe COVID-19 patients [160,161].

## 4. Conclusions

Large amounts of bacteria and viruses are found in the environment. Some of them, called pathogens, can be the cause of severe illnesses or even death. So far, about 1400 human pathogens have been characterized [1]. Pathogens may enter a human organism in many different ways, generally through the consumption of contaminated food and water, or by dust, droplets, and aerosols. Straightforward transmission of zoonosis requires contact between animal hosts and people. In addition, human-to-human transmission is possible, for example, by contact with bodily fluids or contaminated surfaces [162,163]. Pathogens cause diseases using a number of mechanisms, e.g., toxin production, rapid replication, or induction of an excessive immune response [1]. Numerous bacteria develop a variety of responses to harmful conditions, such as heterogeneity in population, growth regulation, and proteolytic system. The widespread use of antibiotics in medicine has resulted in the phenomenon of bacterial resistance to antibiotics. The first MRSA strains were reported in 1961 and their prevalence significantly increased in 1980s. Moreover, according to the 2017 CDC report, 30–50% antibiotic therapies were inappropriate or unnecessary. Importantly, the number of resistant bacteria has increased in recent years, whilst the advancement in production of new antibiotics is limited [92,99,164,165]. Besides the use antibiotics in medicine, antibiotic resistance may also appear for another reason. The active use of antibiotics in agriculture has contributed to the release of antibiotics into the ecosystem. Thanks to their durability, antibiotics have been detected in surface water, which could be the cause of bacterial antibiotic resistance, and can also influence the evolution of microbe structures. Because antibiotic-resistant bacteria pose a serious threat to the health and life of the human population, WHO has announced a priority list for research and development of new antibiotics for antibiotic-resistant bacteria [166,167]. 

A significant problem with viruses is their rapid spread. It is estimated, that annually 390 million of people are infected with dengue, which is one of the 20 most ignored tropical diseases [168]. In 2015, roughly 1.3 million of infected people were reported during the Zika virus (ZIKV) outbreak in South America. Since 2016, Zika virus has been actively transmitted to over 95 countries. Recently, ZIKV cases have been reported in India, Bangladesh, Maldives, and Thailand [169]. The influenza virus causes an epidemic among human population every year. The reason may be high virus variability, which is caused by antigenic drift of surface glycoproteins. This allows the virus to avoid pre-existing immunity and common influenza vaccines, which do not provide comprehensive protection [170]. The influenza virus infects 5–15% of the worldwide population annually, causing 3–5 million cases of illness and approximately 500,000 deaths [171,172]. Over the past two decades, three outbreaks of fast spreading, human-to-human transmitted coronavirus have emerged. The first of the outbreaks was Severe Acute Respiratory coronavirus (SARS-CoV), which is the cause of the infectious disease called SARS. The SARS epidemic in Guangdong Province, China in 2002/2003 led to 8437 laboratory-confirmed cases and 813 mortalities in 29 countries on 5 continents. In addition to mortality, the epidemic resulted in a number of other consequences, including panic, restrictive quarantine conditions, or travel and touristic restrictions [173,174]. As a result of more than 300 genome sequences of SARS-CoV in bats, civets, and humans, it was proposed that the epidemic in 2002/2003 was an effect of multiple recombination events from several ancestors of SARS-CoV [175]. The Middle East Respiratory Syndrome coronavirus (MERS-CoV) first emerged in the Kingdom of Saudi Arabia in 2012. In 2015, the virus occurred in South Korea. Until December 2019, over 2499 infected people were confirmed from 27 countries with 37% mortality [176,177]. In December 2019, a novel coronavirus named SARS-CoV-2 appeared, exhibiting a high pandemic potential. In the first half of February 2020, 45,157 cases from 28 countries/region were reported. Moreover, 1115 of them resulted in death. Over the months, the outbreak spread across the world. The newest report (28 October 2020, European Centre for Disease Prevention and Control) concerning the SARS-CoV-2 situation worldwide showed the number of positive cases reached 44,052,388. Among them, 1,168,076 were fatal. In January 2020, after the outbreaks of Ebola in the Democratic Republic of Congo (2019), Zika (2016), Ebola in West Africa (2014), polio (2014), and H1N1 (2009), WHO announced the SARS-CoV-2 epidemic as the sixth worldwide public health emergency [178,179,180].

Despite significant development of civilization, technology, and medicine, the human population is still exposed to many diseases caused by various pathogens. Infectious diseases remain a major cause of mortality worldwide. Therefore, to reduce the risk of rapid, widespread diseases and deaths caused by them, it is necessary to raise global awareness of health hazards posed by pathogens (including how they spread and treatments). Special attention should also be paid to the drugs used and methods applied for rapid identification of pathogens.

## Figures and Tables

**Table 1:** Summary of the most hazardous pathogens for public health.

Pathogen		Fatality Rate	Human to Human Transmission	Treatment	Vaccine Availability	References
Bacteria	*Bacillus anthracis*	Cutaneous anthrax: 1–20%. Gastrointestinal anthrax: 25–60%. Inhalational anthrax: 86–89%.	No	Antibody-based antitoxin, antibiotic (ciprofloxacin or doxycycline), supportive care.	BioThrax	[5,6,7,8]
	*Yersinia pestis*	Septicemic plague: ~50%. Bubonic plague: 40–70%. Pneumonic plague: 50–100%.	Yes (pneumonic plague)	Antibiotic (streptomycin, tetracyclines, and chloramphenicol), supportive therapy.	No	[9,10,11,12]
	*Francisella tularensis*	Up to 30%	No	Antibiotic (tetracyclines, streptomycin, gentamycin).	No	[13,14,15]
	*Staphylococcus aureus*	10–30%	Yes	Antibiotic (vancomycin or daptomycin, telavancin, ceftaroline).	No	[16,17,18,19]
Virus	Ebola viruses	40–90%	Yes	Supportive treatment (fluid resuscitation, correcting electrolyte imbalances, treating secondary infections, medication to support blood pressure, reduce vomiting and diarrhea).	Ervebo	[20,21]
	Influenza viruses	0.1%	Yes	Antiviral medication (oseltamivir, zanamivir, peramivir, baloxavir marboxil), supportive treatment.	The composition of vaccines is reviewed annually and updated as necessary to match circulating influenza viruses.	[22,23]
	Severe acute respiratory syndrome coronavirus (SARS-CoV-2)	0.53–0.82%	Yes	Supportive treatment (oxygen therapy, intravenous fluid infusion).	No	[24,25,26,27]
